# Lung Cancer in Patients of African Descent: A Transcontinental Review of Epidemiology, Disparities, Outcomes, and Opportunities for Equity in Africa, North America, South America, and the Caribbean

**DOI:** 10.1016/j.jtocrr.2025.100887

**Published:** 2025-08-06

**Authors:** Elvis Obomanu, Colton Jones, Verna Vanderpuye, Nazik Hammad

**Affiliations:** a Department of Internal Medicine, Jefferson-Einstein Hospital, Philadelphia, Pennsylvania; b Sidney Kimmel Medical College, Thomas Jefferson University, Philadelphia, Pennsylvania; c Department of Internal Medicine, Jefferson-Einstein Hospital, Philadelphia, Pennsylvania; d National Center for Radiotherapy, Oncology, and Nuclear Medicine, Korle Bu Teaching Hospital, Accra, Ghana; e Division of Hematology-Oncology, St Michael’s Hospital, Toronto, Ontario, Canada

**Keywords:** Lung cancer, African descent, Black people, Transcontinental disparities

## Abstract

Lung cancer in people of African descent is characterized by transcontinental disparities driven by epidemiologic heterogeneity, systemic inequities, and unequal access to health care. Globally, lung cancer incidence and mortality rates vary; however, underdiagnosis and late-stage presentation in low- and middle-income countries obscure the true prevalence of lung cancer because of limited cancer registries and diagnostic infrastructure. In Africa, most patients with lung cancer present at an advanced stage, primarily because of health illiteracy, misdiagnosis, delayed referrals, and inadequate treatment infrastructure. Although tobacco smoking remains a dominant risk factor worldwide, African populations are disproportionately exposed to environmental and occupational hazards, which substantially elevate their lung cancer risk. In North America, Black people experience disproportionately poor outcomes, including lower rates of lung cancer screening, early diagnosis, surgical intervention, and higher mortality rates compared with their White counterparts. In the Caribbean and South America, Black people continue to face racial infrastructural constraints, racial inequities, and elevated exposure to environmental and occupational carcinogens. Systemic barriers perpetuate these disparities, including limited access to screening, genomic testing, and guideline-concordant therapies.

Achieving equity in lung cancer outcomes requires strategic initiatives, including the expansion of lung cancer registries in Africa, the Caribbean, and South America, to inform evidence-based interventions. Urgent national and international measures focused on prevention and care for populations of African descent, implementing robust tobacco control policies, addressing systemic and racial inequities, and strengthening health care systems to report and manage lung cancer efficiently are essential steps toward reducing disparities. A transcontinental collaborative approach that includes establishing lung cancer research consortia is vital to share best practices in screening protocols, optimize early detection strategies and treatment, and advocate for policy reforms that address the global burden of lung cancer in populations of African descent.

## Introduction

Lung cancer remains a pervasive global health threat, accounting for one in five cancer deaths worldwide, with 2.5 million new cases and 1.8 million deaths reported in 2022.^1^ Despite declining incidence and mortality rates in high-income non-African populations, the lung cancer burden is increasing in Africa, the Caribbean, and the Americas.^1^^,^^2^

The persistently high mortality rate in these regions is attributed to inadequate targeted screening,^3^ socioeconomic disparities,^4^ late presentation,^5^ and lack of access to advanced therapies.^6^ A significant knowledge gap exists regarding lung cancer in African-descent populations, particularly in Africa, North America, South America, and the Caribbean. The Global surveillance of trends in cancer survival 2000-2014 (CONCORD-3) study revealed disparities in cancer registry participation, impacting care and resource allocation.^7^ Historical neglect of African-descent populations in lung cancer research, driven by socioeconomic inequality and racism, exacerbates these disparities.^8^, ^9^, ^10^

This review aims to discuss transcontinental disparities, genetic and environmental drivers, and existing gaps in lung cancer care among African-descent populations, informing strategies for equitable care.

## Methods

This traditional narrative review was conducted using an extensive search of multiple online electronic databases (PubMed, Ovid, EMBASE, Cochrane Central Register of Controlled Trials, and Google Scholar) from January 1, 2012 to February 2025.

We conducted a mixed-method literature search for retrospective studies, prospective studies, clinical trials, and literature reviews that discussed transcontinental disparities, genetic and environmental drivers, and existing gaps in lung cancer care among populations of African descent. Our search keywords were: “lung cancer in African descent,” “lung cancer in Blacks,” lung cancer in Latin America,” lung cancer in the Caribbean,” “lung cancer epidemiology in Blacks,” “lung cancer in Blacks incidence and mortality,” “lung cancer treatment in Africans OR Blacks.” We also evaluated the reference lists of many review articles to identify additional studies. Case reports and case series were excluded. In addition, we excluded articles written in languages other than English and those on nonhuman subjects.

## Epidemiology of Lung Cancer Across Africa and the African Diaspora

### Africa

**Figure 1:**
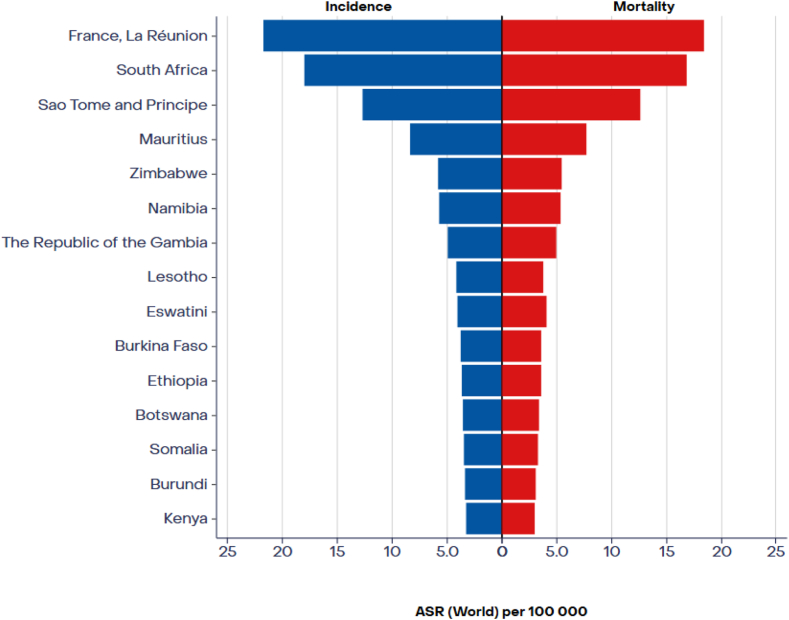
ASR (world) (per 100,000) incidence and mortality, both sexes, in 2022, trachea, bronchus, and lung. Sub-Saharan Africa Hub (Top 15). Adapted from: https://gco.iarc.who.int. ASR, age-standardized rate.

The age-standardized incidence and mortality rates of lung cancer in Africa (Fig. 1) exhibit significant geographic variability. Notably, North African men exhibit the highest incidence rate (20.6/100,000), whereas West African women had the lowest incidence (1.6/100,000). Conversely, Southern African men had the highest mortality rate (23.7/100,000). In contrast, West African women had the lowest mortality rate (1.5/100,000^11^). This disparate pattern is attributed mainly to the tobacco epidemic in the region.^12^ Furthermore, urbanization has increased exposure to environmental and occupational hazards, and tobacco products, thereby contributing to the elevated incidence of lung cancer in urban areas compared with rural regions.^13^ Consistent with global trends, NSCLC is the most prevalent subtype in Africa, with squamous cell carcinoma being more common in Northern Africa and adenocarcinoma in Southern Africa, reflecting the strong association between smoking habits, environmental carcinogens, and lung cancer histologic subtypes.^14^

However, it is essential to acknowledge the challenges posed by underdiagnosis and the paucity of cancer registries, which compromise the accuracy of lung cancer reporting in Africa.^15^ In addition, lung cancer mortality rates in Africa may be underestimated because of the prolonged latency period between tobacco smoking and disease onset, coupled with low life expectancy resulting from competing factors such as communicable diseases^16^

### North America

**Figure 2:**
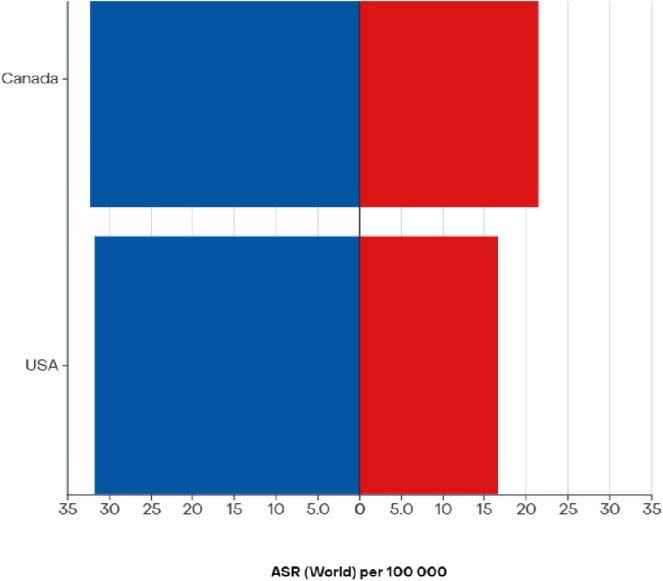
ASR (world) per 100,000, incidence, and mortality, both sexes, in 2022, trachea, bronchus, and lung. North America. Adapted from: https://gco.iarc.who.int. ASR, age-standardized rate.

In North America, the United States and Canada have well-established cancer registries. Still, a significant limitation exists in Canada because of its color-blind approach, which precludes data disaggregation by race.^17^ Consequently, insights into lung cancer disparities among African-descent populations in Canada are hindered. In contrast, data from the United States reveal a disproportionate burden of lung cancer among African American and Black individuals, characterized by the lowest survival rates and highest mortality rates (Fig. 2) among all racial groups.^2^ Notably, Black men and women in the United States are more likely to receive a lung cancer diagnosis at a younger age compared with their White counterparts.^18^ Furthermore, 5-year survival outcomes are significantly lower for African Americans (20%) compared with Whites (22%), with this survival disparity exacerbated in cases of localized disease (55% versus 60%).^2^

### Caribbean and South America

**Figure 3:**
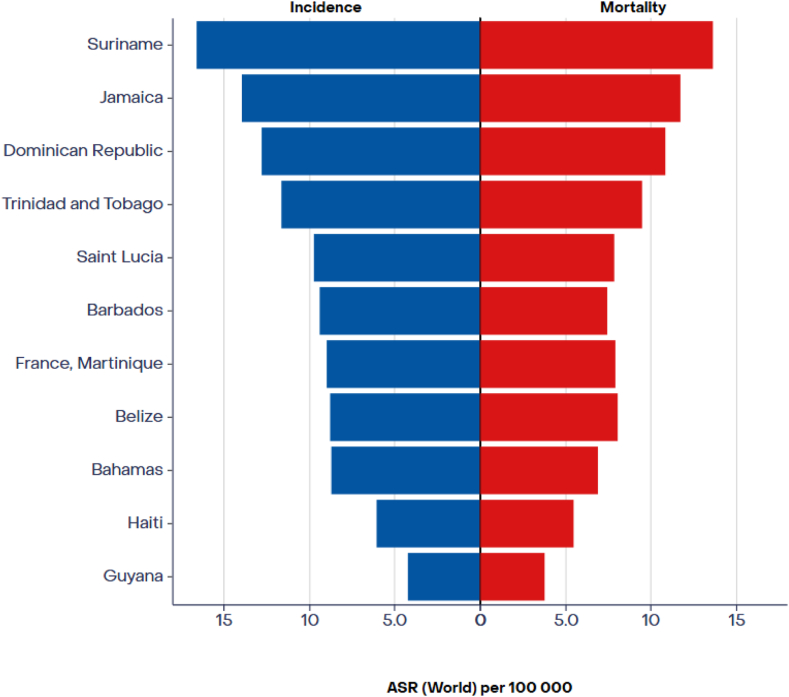
ASR (world) per 100,000, incidence and mortality, both sexes, in 2022, trachea, bronchus, and lung, Caribbean Hub. Adapted from: https://gco.iarc.who.int. ASR, age-standardized rate.

**Figure 4:**
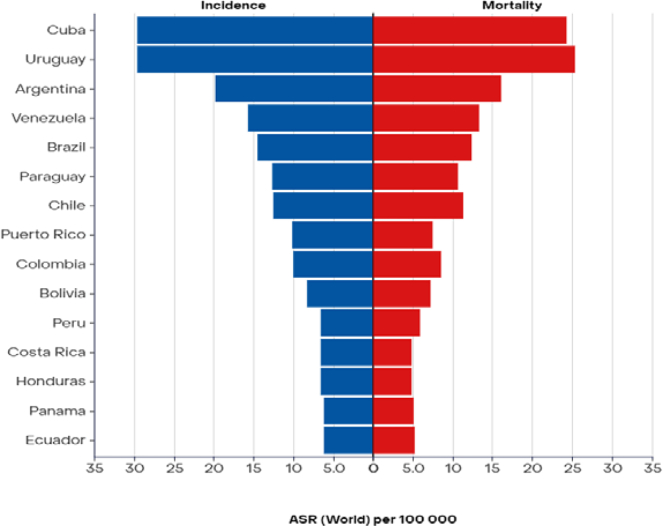
ASR (world) per 100,000, incidence and mortality, both sexes, in 2022, trachea, bronchus, and lung, Latin America Hub (Top 15).Adapted from: https://gco.iarc.who.int. ASR, age-standardized rate.

A significant limitation in cancer reporting in the Caribbean and South America is the lack of participating cancer registries, hindering accurate epidemiologic studies and racial disparities analysis.^1^^,^^7^ The available data from 2022 indicate a substantial burden of lung cancer in these regions, with 3366 reported cases and 2900 deaths in the Caribbean, exhibiting a male predominance in both incidence and mortality rates (Fig. 3). Jamaica and Haiti, with significant populations of African descent, collectively reported 1018 incident cases and 791 deaths from lung cancer in 2022. In South America, 91,857 incident cases and 78,559 deaths from lung cancer were recorded in 2022 (Fig. 4), although these data were not disaggregated by racial groups.^19^ A comparative study in Sao Paulo, Brazil, revealed that Black people experience higher cancer mortality rates compared with other ethnic groups.^20^

## Risk Factors

Tobacco smoking is a paramount risk factor for lung cancer development,^21^ and the tobacco epidemic disproportionately affects low- and middle-income countries (LMICs) because of lenient tobacco policies and targeted marketing to racialized minorities.^22^, ^23^, ^24^ The histologic subtypes of lung cancer prevalent in various continents reflect regional smoking habits.^14^ Furthermore, exposure to secondhand smoke increases lung cancer incidence,^25^ with rising rates observed in Africa.^26^ Racialized minorities, particularly non-Hispanic Blacks in the United States, are more likely to be exposed to secondhand smoke because of socioeconomic disparities.^27^ These populations also exhibit higher rates of menthol cigarette smoking and nicotine dependence.^28^^,^^29^ Notably, Black smokers exhibit differential intake of toxic volatile compounds such as acrolein, acrylonitrile, and ethylene oxide in tobacco, increasing their lung cancer risk.^30^ In addition, Black patients exhibit higher cotinine levels, a metabolite of nicotine, because of reduced glucuronidation, which is associated with an increased risk of lung cancer.^31^^,^^32^

In Africa, the widespread use of biomass fuels and coal for cooking leads to significant indoor air pollution.^33^ This exposure to carcinogenic substances increases lung cancer risk.^34^ In Africa, this risk is borne disproportionately by women and young girls who have significantly more exposure to household air pollutants as they spend more time cooking and being indoors,^35^ highlighting an essential contribution to the observed steady increase in lung cancer in African women. Studies from several African countries indicate that biomass fuels remain the primary source of household energy, particularly in rural and periurban areas. For instance, 77% of Cameroonian households use only firewood, and just 13.8% of Ghanaian women of reproductive age use liquefied petroleum gas. Adoption of cleaner fuels is strongly linked to socioeconomic status, education, and urban living.^36^, ^37^, ^38^ Furthermore, exposure to diesel exhaust fumes and urban traffic pollution increases lung cancer risk in these vulnerable populations.^39^^,^^40^

A Canadian study reported that wildfire exposure has also been linked to increased lung cancer risk,^41^ with socioeconomic disparities exacerbating exposure among Black populations.^42^ Occupational exposures significantly contribute to lung cancer risk, with certain industries and Black populations disproportionately affected.^43^ Notably, studies have reported that workers in the Caribbean sugarcane farming, rum production, painting, and motor vehicle repair industries face elevated lung cancer risks.^44^ Similarly, African miners exposed to carcinogenic substances such as silica dust, beryllium, nickel, radon, and asbestos are at increased risk of developing lung cancer.^45^ In the United States, African Americans are more likely to experience occupational exposure to asbestos, silica, and fumes, thereby increasing their lung cancer risk.^46^ These findings underscore the need for targeted occupational health interventions and policies to mitigate lung cancer disparities in vulnerable populations.

Individuals with human immunodeficiency virus (HIV) infection face a significantly elevated risk of lung cancer, with a two to sevenfold increased likelihood compared with the general population, as reported by Molto et al.^47^ The elevated risk observed in this population can be attributed to a trifecta of factors: severe immunocompromise, chronic pulmonary inflammation, and the disproportionately high prevalence of smoking among individuals with HIV. Dalyan et al.^48^ observed that HIV-associated lung cancer disproportionately affects patients of African descent. This disparity is further exacerbated by the higher prevalence of HIV infection among individuals of African descent,^49^^,^^50^ thereby contributing to an increased risk of lung cancer development in this population.

## Molecular Biology and Genomics

Disparities in biomarker testing for patients of African descent with lung cancer have resulted in significant knowledge gaps and limited generalizability of novel therapies to this population. Biomarker testing for lung cancer in Africa and the Caribbean is low and not usually the standard of care because of funding and infrastructural constraints^51^, ^52^, ^53^, ^54^ Compared with their White counterparts, African-descent patients are less likely to undergo biomarker testing^55^ despite exhibiting distinct molecular profiles, including higher tumor mutational burden and reduced targetable mutations.^56^ Notably, these patients have a lower prevalence of EGFR, ALK, and ROS-1 mutations,^57^^,^^58^ which has implications for the effectiveness of novel targeted therapies. Furthermore, Jiagge et al.^59^ reported that patients of African descent with lung cancer have depleted KRAS G12C and EGFR L858R mutations, predictive of response to anti-KRAS therapies and EGFR tyrosine kinase inhibitors, thereby diminishing their response to targeted therapies.

Genomic and molecular analyses have revealed distinct characteristics of lung cancer in African American patients, contributing to poor prognosis and treatment resistance. Mutations in STK11, a key regulator of cellular metabolism and apoptosis, are more prevalent in Black patients and are associated with poorer outcomes.^60^ Furthermore, STK11 mutations drive resistance to programmed cell death protein 1 (PD-1) inhibitors in NSCLC.^61^ African Americans with lung adenocarcinoma also exhibit an increased prevalence of PTPRT and JAK2 mutations,^62^ which can lead to loss of programmed death-ligand 1 expression and resistance to anti–programmed death-ligand 1 therapy.^63^ Unique inflammatory protein signatures, including interleukin-10, interleukin-15, tumor necrosis factor–β, and monocyte chemotactic protein–4, are associated with lung cancer in Black patients.^64^ In addition, Brown et al.^65^ reported that tumors from Black patients are more likely to exhibit whole-genome duplications, a genomic event linked to enhanced aggressiveness and metastasis, compared with tumors from White patients. These findings highlight the importance of considering the distinct molecular and genomic profiles of lung cancer in African American patients to develop effective treatment strategies.

## Clinical Presentation and Outcomes

African Americans and Black individuals experience the highest cancer mortality rates and lowest survival rates among all racial groups, with lung cancer being a significant contributor to these disparities. The 5-year survival rate for lung cancer is substantially lower in African Americans (20%) compared with whites (22%), with a more pronounced disparity in localized disease (55% versus 60%).^2^ The CONCORD-3 study revealed a striking gap in age-standardized survival rates between Africa, the Caribbean, and South America (10%–20%) and the United States and Canada (20%–30%).^7^ These poor outcomes are attributed to delayed diagnosis, reduced access to curative treatments for early-stage lung cancer, and inadequate stage-appropriate chemotherapy in Black patients.^66^, ^67^, ^68^ It is imperative to note that although sub-Saharan Africa and Northern Africa struggle with late diagnosis and poor outcomes, sub-Saharan Africa is disproportionately affected by weak health systems, limited diagnostics, and higher comorbidity. In contrast, Northern Africa’s challenges are more related to rising tobacco use and improving but still limited cancer care infrastructure.^15^^,^^69^ Mitigating these disparities is essential for achieving equitable cancer outcomes and addressing health inequities that disproportionately affect African American and Black communities.

In LMICs, patients of African descent face significant barriers to effective cancer care, including a lack of localized treatment guidelines, limited access to novel chemotherapeutic agents, and inadequate health care infrastructure.^3^^,^^4^ The impact of natural disasters and conflict on cancer care in these regions further exacerbates poor outcomes, disproportionately affecting Black patients.^70^^,^^71^ The reliance of cancer care in Africa, the Caribbean, and South America on funding from external sources, such as the United States, renders these programs vulnerable to budgetary fluctuations and withdrawal of support, compromising patient outcomes.^72^ In addition, the high prevalence of comorbidities among Black patients contributes to disparate cancer survival outcomes, with a worse prognosis compared with other racial groups.^44^^,^^73^ Addressing these systemic challenges is critical to improving cancer outcomes and reducing health inequities in African descendant populations.

## Systemic and Cultural Barriers to Care

The U.S. National Lung Screening Trial and the Dutch-Belgian Randomized Lung Cancer Screening Trial (NELSON study) have reported the efficacy of low-dose computed tomography screening in reducing lung cancer incidence and mortality.^74^^,^^75^ However, this life-saving intervention is inaccessible mainly in Africa, the Caribbean, and Latin America, where formal lung cancer screening programs are lacking.^15^^,^^76^^,^^77^ Furthermore, racial disparities persist in the United States, where African Americans, despite being at increased risk for lung cancer, are less likely to undergo screening compared with their White counterparts.^78^ A study of U.S. veterans revealed that Black veterans had 34% lower odds of receiving lung cancer screening compared with their White counterparts.^79^ Treatment disparities also persist, with Black patients more likely to forgo or discontinue treatment because of financial constraints and inadequate insurance coverage.^80^^,^^81^ Blacks have been found to wait longer for curative intent lung surgeries compared with other racial groups.^82^ Access to surgery, radiotherapy, and cancer medicines, including immunotherapy and targeted therapies, remains a significant challenge for the treatment and palliation of lung cancer patients in Africa and Diasporic Africans in LMIC. The underrepresentation of African ancestry patients in clinical trials, genomic studies, and biomarker testing worsens these disparities, compromising outcomes.^8^^,^^83^ Historical events, such as the Tuskegee syphilis study, have fostered mistrust, further hindering participation in research among this population.^84^

The lack of clinical trials in LMIC^9^^,^^85^ results in a significant lag in accessing innovative lung cancer treatments and therapeutics for populations in these regions. This disparity is further accentuated by inadequate financing of health care systems, leading to limited availability of essential cancer services, including chemotherapy.^86^ In addition, systemic barriers, such as health illiteracy, poor health-seeking behaviors, and inefficient referral systems, disproportionately impede cancer care for Black patients, perpetuating health inequities.^3^

## Current Initiatives and Persistent Gaps

The International Agency for Research on Cancer's Global Initiative for Cancer Registry Development, launched in 2011, has catalyzed efforts to enhance cancer reporting systems in Africa through the African Cancer Registry Network.^15^ Similarly, the Caribbean Cancer Research Institute is tackling cancer disparities in the region by means of research, implementation, and advocacy. These initiatives aim to improve cancer outcomes and reduce disparities in vulnerable populations. The Human Heredity and Health in Africa initiative, launched in 2012, has significantly addressed genetic research inequities in African populations. By conducting whole-genome sequencing in 32 African countries, the Human Heredity and Health in Africa initiative seeks to bridge the gap in global health and genetic research, enabling the development of novel therapies tailored to African populations. In the sequel to the Accountability for Cancer Care through Undoing Racism and Equity (ACCURE) trial,^87^ quality improvement initiatives have used an equity dashboard method targeting wait times in lung cancer surgery in African American patients, which has resulted in improved timely lung cancer surgery for Black and White patients with early NSCLC.^88^ Furthermore**,** lung cancer programs such as the Multinational Lung Cancer Patient Control Program in Sub-Saharan Africa and Cancer Care Africa are improving access to lung cancer screening, diagnostics, and quality of care in Swaziland, Tanzania, Kenya, and South Africa.

Despite efforts to improve diversity in clinical research, African-descent populations continue to be underrepresented in clinical trials.^6^ Moreover, cancer registries in Africa, the Caribbean, and South America face significant challenges, including inadequate funding and infrastructure, which hinder their ability to provide high-quality, actionable data. This data gap impedes clinical research, policy development, resource allocation, and efforts to address systemic disparities. In addition, in Africa, there is a shortage of cardiothoracic surgeons, radiation, and medical oncologists, hampering cancer care.^3^ The critical role of tobacco in lung cancer development necessitates stringent regulations to reduce consumption. However, tobacco control policies in LMICs remain weak, perpetuating the tobacco epidemic in these regions.^23^ Socioeconomic disparities continue to affect lung cancer screening, diagnosis, and treatment, with underfunding exacerbating the scarcity of diagnostic facilities, chemotherapeutic regimens, and research initiatives.

## Future Directions

Preventive measures are essential in reducing the burden of cancer in Black and African populations. To counter the targeted marketing of tobacco products to these communities, culturally sensitive smoking cessation policies must be developed, addressing the socioeconomic drivers of nicotine addiction. Furthermore, policies should be enacted to mitigate exposure to environmental and occupational carcinogens, which disproportionately affect Black populations. A global partnership is crucial for regulating tobacco use, enhancing occupational safety, and promoting health equity.

To harness the benefits of cancer research for Black populations, it is imperative to integrate genomic studies, biomarker testing, and clinical trial equity, inclusive of participants of African descent. This strategic approach will facilitate the development of novel, genetically tailored therapeutics for this population. Establishing global research consortia comprising Black researchers from Africa and the diaspora has effectively augmented research output. Notable examples include the Men of African Descent and Carcinoma of the Prostate consortium and the Prostate Cancer Transatlantic Consortium, which leverage transcontinental partnerships to enhance prostate cancer prevention, detection, and treatment in men of African descent. Similarly, the African Ancestry Breast Cancer Genetic Consortium, funded by the National Institutes of Health, investigates genetic and biological factors contributing to breast cancer risk in Black women. A comparable consortium focused on lung cancer in Black populations would be instrumental in advancing knowledge and improving outcomes for this population. The creation of such a collaborative effort would be a timely and beneficial step toward addressing the disparities in lung cancer research and care affecting populations of African descent. Implementing cost-effective, low-dose computed tomography screening programs in Africa, the Caribbean, and South America, and addressing screening disparities in North America, are essential steps toward reducing lung cancer mortality in these populations.

Cancer registries are important sentinels that expose vulnerabilities in diagnosis, treatment, and care delivery. Enhancing their capacity and accuracy is essential for shifting from a reactive to a proactive approach to cancer control. By upgrading and standardizing cancer registries, health care systems can better address systemic disparities, inform resource allocation, design targeted interventions, and support translational research. Robust cancer registries are a cornerstone for achieving equitable cancer care and improving outcomes for diverse populations.

In conclusion, lung cancer in individuals of African descent is a complex biosocial entity shaped by the interplay of biological and social factors. Distinct genetic and epigenetic profiles, coupled with environmental injustices, occupational exposures, and permissive tobacco regulations, have converged to drive the disproportionate burden of lung cancer in this population. Furthermore, social determinants, including systemic racism and poverty, worsen outcomes, highlighting the need for a multifaceted approach. A transcontinental collaboration spanning the Americas, Africa, and the Caribbean is imperative to dismantle the entrenched disparities in lung cancer outcomes affecting individuals of African descent.

## CRediT Roles

**Elvis Obomanu**: Conceptualization, Methodology, Validation, Data Curation, Writing – original draft, Writing – review & editing, Supervision. **Colton Jones**: Methodology, Validation, Writing – original draft, Writing – review & editing. **Verna Vanderpuye**: Writing – original draft, Writing – review & editing. **Prof Nazik Hammad**: Conceptualization, Methodology, Validation, Writing – review & editing, Supervision.

## Disclosure

The authors declare no conflict of interest.
